# Assessing Donor Site Morbidity and Impact on Quality of Life in Free Flap Microsurgery: An Overview

**DOI:** 10.3390/life15010036

**Published:** 2024-12-30

**Authors:** Florin-Vlad Hodea, Cristian-Sorin Hariga, Eliza-Maria Bordeanu-Diaconescu, Andrei Cretu, Catalina-Stefania Dumitru, Vladut-Alin Ratoiu, Ioan Lascar, Andreea Grosu-Bularda

**Affiliations:** 1 Department 11, Discipline Plastic and Reconstructive Surgery, Bucharest Clinical Emergency Hospital, University of Medicine and Pharmacy Carol Davila, 050474 Bucharest, Romania; florin-vlad.hodea@drd.umfcd.ro (F.-V.H.); andrei.cretu@drd.umfcd.ro (A.C.); catalina-stefania.dumitru@rez.umfcd.ro (C.-S.D.); vladut-alin.ratoiu@rez.umfcd.ro (V.-A.R.); ioan.lascar@umfcd.ro (I.L.); andreea.grosu-bularda@umfcd.ro (A.G.-B.); 2 Clinic of Plastic Surgery and Reconstructive Microsurgery, Clinical Emergency Hospital of Bucharest, 014461 Bucharest, Romania; eliza.diaconescu@umfcd.ro

**Keywords:** free flap, microsurgery, donor site, morbidity, quality of life

## Abstract

Donor site morbidity remains a significant concern in free flap microsurgery, with implications that extend beyond immediate postoperative outcomes to affect patients’ long-term quality of life. This review explores the multi-faceted impact of donor site morbidity on physical, psychological, social, and occupational well-being, synthesizing findings from the existing literature. Particular attention is given to the functional limitations, sensory deficits, aesthetic outcomes, and chronic pain associated with commonly utilized free flaps. Advancements in surgical techniques, including nerve-sparing and muscle-sparing methods, as well as innovations, like perforator flaps, have demonstrated the potential to mitigate these morbidities. Furthermore, the integration of regenerative medicine strategies, such as stem cell therapy and fat grafting, and technological innovations, including virtual reality rehabilitation and biofeedback devices, has shown promise in enhancing recovery and minimizing long-term complications. Despite these advances, challenges persist in standardizing QoL assessments and optimizing donor site management. This review emphasizes the need for a holistic, patient-centered approach in reconstructive microsurgery, advocating for further research to refine current strategies, improve long-term outcomes, and develop robust tools for QoL evaluation. By addressing these gaps, reconstructive surgeons can better align surgical objectives with the comprehensive well-being of their patients.

## 1. Introduction

Free flap microsurgery has changed the field of reconstructive surgery, offering innovative and effective solutions for addressing intricate defects that arise from a variety of sources, including traumatic injuries, oncological resections, or congenital anomalies. These types of surgeries consist of the transfer of vascularized tissue from a donor site, often located at a distance from the area requiring reconstruction, to a recipient site, thereby resulting in the restoration of both aesthetic form and function. The versatility of free flap procedures allows surgeons to utilize a range of tissue types, including skin, muscle, and bone, tailored to the specific needs of the patient and the nature of the defect. This adaptability not only enhances the outcomes of reconstructive efforts but also significantly improves the quality of life for patients, enabling them to regain their physical appearance and functionality [1].

Donor site morbidity refers to the complications and adverse effects that may arise at the anatomical location from which tissue is harvested during free flap microsurgery. These procedures are crucial in the realm of reconstructive surgery; however, it is essential to address the complex challenges associated with donor site morbidity to optimize patient outcomes and improve post-surgical quality of life (QoL) [2]. A primary concern regarding donor site morbidity is the potential for physical complications, which may manifest as pain, scarring, and functional limitations in the area from which tissue is taken. Beyond the physical consequences, donor site complications can also lead to psychological effects. The combination of pain, altered body image, and reduced functionality may contribute to feelings of anxiety, depression, or dissatisfaction with one’s physical appearance [3]. Moreover, the length of recovery associated with donor site morbidity can extend the rehabilitation process, subjecting patients to prolonged discomfort and necessitating further medical interventions or physical therapy. Such extended recovery periods may further impact emotional well-being and social interactions, potentially leading to isolation from support networks and adversely affecting mental health [4]. While free flap procedures are essential for restoring anatomical form and functional abilities, the implications of donor site morbidity require careful attention. These complications have significant repercussions for post-surgical QoL, affecting both physical function and psychological well-being [5].

The primary aim of this narrative review is to assess QoL outcomes and evaluate morbidity associated with various donor sites utilized in free flap microsurgery. By focusing on the specific complications and challenges that arise from different anatomical harvest locations, this review seeks to provide a comprehensive analysis of how donor site morbidity impacts patients’ overall well-being, such as pain and functional limitations, as well as psychological effects, including body image concerns and emotional distress. This targeted assessment will not only elucidate the relationship between donor site morbidity and QoL but also identify best practices and potential strategies for minimizing adverse outcomes. Ultimately, the findings of this review aim to inform clinicians and healthcare providers, fostering a deeper understanding of the importance of donor site considerations in enhancing the overall recovery experience for patients undergoing free flap procedures.

## 2. Materials and Methods

This study was elaborated as a narrative review aimed at synthesizing the existing literature on the relationship between free flap procedures and their associated donor site morbidity, as well as the subsequent impact on quality of life. To achieve a comprehensive understanding, we conducted a search across academic databases, including Google Scholar, Web of Science, and PubMed. Our search strategy centered on the keywords “free flap” and “microsurgery” and combined keywords, such as “donor site morbidity” and “quality of life”. We focused our evaluation on original research studies that aligned closely with our specific area of interest, ensuring that the selected articles provided relevant insights and data. Furthermore, we included studies published up to the year 2024.

## 3. Discussion and Literature Review

In free flap microsurgery, each defect presents anatomical and functional characteristics that may require a specific flap to be used for coverage based on functional and aesthetic considerations. Flaps harvested from different donor sites present unique benefits, limitations, and potential risks, impacting suitability for certain surgical goals. Certain reconstructive options are favored for their versatility in soft tissue coverage, especially in areas like the head, neck, and extremities, due to their reliable tissue volume and ease of contouring. Others, such as breast reconstruction, are highly valued in reconstructive applications that require significant fat volume and preservation of muscle, making them particularly suitable for areas needing both functional and aesthetic outcomes. Beyond the functional advantages and practical considerations, donor site selection must also weigh the potential for morbidity and its impact on QoL. Donor site morbidity, including functional limitations, aesthetic concerns, sensory deficits, and chronic pain, can vary significantly depending on the anatomical site and flap type.

### 3.1. Commonly Used Flaps in Microsurgery

Microsurgical reconstruction is required in complex situations, primarily involving significant tissue defects that cannot be adequately addressed by simpler surgical methods. The selection of specific flaps for various surgical situations is influenced by multiple factors, including patient characteristics, the specific surgical site, and the nature of the defect or trauma. In breast reconstruction, factors such as body mass index (BMI) and age play a significant role. For instance, the profunda artery perforator, lumbar artery perforator, and superior gluteal artery perforator flaps are particularly advantageous for slim patients with lower BMIs, as they maintain sufficient thickness even when BMI decreases, unlike other flaps that may be affected by age and BMI variations [6]. For head and neck cancer reconstruction, the choice of flap is determined by the region of the defect and the specific functional and aesthetic requirements, with advances in techniques allowing for sensate free tissue transfer and dental rehabilitation [7]. Finally, in the reconstruction of complex upper extremity trauma, factors such as skin thickness, texture, color, and the surgeon’s experience are crucial, along with the timing of the reconstruction to ensure optimal functional recovery [8]. Oncological resections from simple, rare tumors and soft tissue tumors to complex multidimensional ones, such as in the head and neck, and other areas, like the breast, trunk, and extremities, also frequently require a step-wise reconstructive ladder approach from simple closure to microsurgical reconstruction in order to manage large defects and restore function and contour [9,10]. Across these contexts, the choice of flap is critical for successful outcomes, with various flaps, as seen in Table 1, being commonly employed. Overall, the need for microsurgical reconstruction arises from the necessity to address complex defects that require precise and durable solutions to restore function and aesthetics [11,12].

The ALT flap is one of the most versatile and commonly utilized free flaps in reconstructive microsurgery. It is primarily supplied by the descending branch of the lateral circumflex femoral artery, which provides robust vascularity crucial for flap survival. The ALT flap can be designed as chimeric or simple, fasciocutaneous or myocutaneous, and thick or thin, depending on the reconstructive needs of the defect. It typically consists of skin, fascia, and, if necessary, muscle, which makes it adaptable for a range of applications. Surgeons can modify its size and contour to suit various reconstructive requirements, which makes it a highly reliable option for complex defects. Additionally, because the ALT flap has a long and sizable vascular pedicle, it allows for greater reach and flexibility in positioning. All of these considerations make it the ideal flap, adaptable to most situations. However, the ALT flap presents certain challenges. The vascular anatomy of the flap can vary considerably among patients, which may complicate the dissection and design process. Variations in perforator location, size, and number require careful preoperative planning, often involving Doppler ultrasound or imaging studies to map the vascular supply accurately [47,48,49].

The DIEP flap, supplied by perforating branches of the deep inferior epigastric artery, is a popular choice for soft tissue reconstruction, particularly in breast reconstruction, due to its ability to a significant adipocutaneous flap volume without sacrificing the underlying muscle. This muscle-sparing flap reduces donor site morbidity compared to older techniques, such as the TRAM flap, making it a preferred option among reconstructive surgeons. The main advantages of the DIEP flap include its aesthetic contour and the preservation of abdominal muscle strength, which allows for improved functional outcomes and faster recovery. Additionally, the flap’s large volume of available soft tissue allows for a natural appearance in breast reconstruction, closely matching the patient’s body profile [50,51,52]. However, the DIEP flap procedure is technically challenging compared to the alloplastic conventional reconstruction of the breast. The DIEP flap is, however, preferred compared to other autologous reconstruction variants, especially in radiated breast oncological resections [53,54].

The RFFF is a versatile option in reconstructive surgery that is frequently used for head and neck defects and is also used in other areas where a thin, pliable flap is needed. Supplied by the radial artery, the RFFF offers a robust blood supply and allows for a long vascular pedicle, providing flexibility in positioning the flap. One of the main benefits of the RFFF is its pliability, which enables precise shaping and adaptation to complex anatomical areas, especially for the pharynx, nose, lip, palate, and mouth floor. Additionally, it is relatively straightforward to harvest, with a consistent vascular anatomy that minimizes intraoperative unpredictability. However, the RFFF does come with some drawbacks, particularly the visible donor site on the forearm, which can lead to aesthetic concerns and potential sensory deficits. Moreover, its harvest can lead to weakness in radio-carpal joint motion [55,56,57].

The fibula free flap is a gold standard in reconstructive surgery for osseous defects, particularly in mandibular, maxillary, and long bone reconstructions. Its versatility comes from its unique ability to provide both bone and soft tissue, enabling surgeons to reconstruct complex bone defects needing large segment reconstruction. The flap is typically harvested with the peroneal artery as its primary blood supply, allowing it to support extensive bone segments while also providing options for skin paddles or muscle from solear or flexor hallucis muscles if soft tissue coverage is needed. A significant advantage of the fibula flap is its durability and strength, which are ideal for load-bearing reconstructions, particularly in the mandible. Additionally, the fibula’s length and shape can be customized, segmented, or contoured, allowing it to adapt to varied defect sizes and shapes. However, the harvest of the fibula can be technically demanding and may lead to donor site morbidity, including potential weakness or reduced range of motion in the ankle [58,59,60].

Of great importance is imaging whenever harvesting a free fibula flap. The presence of a single-vessel or dual-vessel arterial supply can significantly impact surgical planning. The study by Apaydın et al. highlights the importance of understanding the vascular anatomy, noting that the fibular artery supplies the proximal and distal portions of the fibular muscles, which is critical information for avoiding complications during graft harvesting [61]. Hölzle et al. provide insight into the perfusion changes post-fibula transfer, noting a significant reduction in blood flow in the peroneal artery region, which could contribute to delayed healing, although these changes were below the threshold of clinical manifestation [62]. Diabetes mellitus, smoking, and peripheral vascular disease are associated with an elevated risk of complications and poor wound healing at the fibula donor site. These conditions can substantially compromise blood flow and the healing process, leading to a higher likelihood of surgical site infections and graft failure [63]. In addition, a systematic review found that full-thickness skin grafts were the most commonly used method for covering fibula flap defects, but they had higher rates of partial and complete necrosis compared to split-thickness skin grafts and flap methods, with the latter showing the lowest complication rates [64].

The LD flap is a well-established option in reconstructive surgery, particularly useful for its large volume and robust blood supply, which make it suitable for extensive soft tissue defects. This flap is supplied by the thoracodorsal artery and can include both muscle and skin, making it ideal for areas requiring significant bulk, such as in the breast, chest wall, and upper extremity reconstructions. The LD flap is frequently used when other options may not provide enough tissue or when local flap options are insufficient. One of the primary benefits of the LD flap is its size and volume, allowing it to cover large defects effectively. Additionally, it is relatively easy to harvest and has a reliable vascular pedicle, which makes it a dependable choice in complex cases. However, its bulk can sometimes be a limitation in terms of contour, and donor site morbidity, such as shoulder weakness, is a potential concern [65,66,67].

The gracilis free flap is commonly used in reconstructive microsurgery for smaller, specialized defects and functional muscle transfers, such as facial reanimation or perineal reconstruction, if pedicled. Supplied by the gracilis branch of the profunda femoris artery, the flap provides a vascularized muscle that can be transferred with or without overlying skin, such as the transverse-upper gracilis, depending on the reconstructive needs [68]. Its thin, elongated shape makes it well suited for linear defects, particularly where bulk is not a primary requirement. One of the primary advantages of the gracilis flap is its minimal donor site morbidity, as harvesting the gracilis muscle has little impact on the functionality of the thigh. This makes it a favorable choice for small- to medium-sized defects that require a dynamic muscle transfer or soft tissue coverage with minimal contour disturbance. However, the gracilis flap is limited in size, and its muscle bulk is insufficient for larger reconstructions. Despite these limitations, it is a reliable option for specific needs, such as facial reanimation and certain lower extremity or perineal reconstructions [69,70,71].

The SCIP flap is harvested from the groin area, supplied by the superficial circumflex iliac artery, and includes skin and fascia, providing thin and pliable tissue ideal for reconstructing small- to medium-sized soft tissue defects. Because the SCIP flap is minimally invasive and does not involve muscle harvest, it offers low donor site morbidity and maintains both functional and aesthetic integrity at the donor site. One of the main advantages of the SCIP flap is its thin, flexible nature, making it suitable for delicate reconstructions in areas such as the head and neck, hand, and extremities where bulkiness could be problematic. Additionally, the donor site scar can be well hidden in the groin crease, which is beneficial for patients concerned about visible scarring. However, due to the smaller and more variable perforator vessels associated with this flap, meticulous planning and careful dissection are essential. Despite these challenges, the SCIP flap has quickly gained popularity for its versatility and low impact on the donor site [72,73,74].

The PAP flap has become a valuable backup option in reconstructive surgery, especially when other donor sites are unavailable or unsuitable for both breast and head and neck cases. Harvested from the posterior thigh and supplied by the profunda femoris artery perforators, the PAP flap provides soft tissue coverage without sacrificing muscle, making it particularly suitable for areas where a moderate amount of bulk is required, such as breast reconstruction. It has the advantage of low donor site morbidity as well as a scar that can be well hidden. The PAP flap has demonstrated reliable outcomes, especially in cases where abdominal tissue is not available or when patients prefer an alternative to abdominal flaps for breast reconstruction [75,76].

The MSAP flap is another useful option, especially for small- to medium-sized defects where thin, pliable tissue is necessary. Supplied by the medial sural artery perforators, this flap is harvested from the posterior calf and offers a thin, flexible tissue composition ideal for head and neck or hand reconstructions. Its relatively hidden donor site and minimal morbidity make it appealing for patients concerned about visible scarring. While the MSAP flap requires meticulous dissection due to the smaller and more variable perforators, it provides an effective backup for areas where bulkier flaps, such as a conventional ALT, may not be suitable [77].

The TDAP flap serves as a muscle-sparing flap and is used in areas such as the chest wall and extremity reconstruction. The TDAP flap, supplied by perforators from the thoracodorsal artery, allows surgeons to transfer a fasciocutaneous flap without harvesting the latissimus dorsi muscle, thus preserving shoulder function. This flap offers flexibility in contour and is useful for moderate-sized defects, though anatomical variability of perforator location can complicate harvest. Although anterior harvesting techniques have been described, usually lateral decubitus harvest is employed, which may complicate intraoperative logistics [36,78,79].

Although these are the most commonly used flaps, there is a wide array of flaps that have specific indications, such as the ulnar forearm flap, or are presented as alternatives to conventional flaps, such as lumbar artery perforator flaps, which are superior and inferior gluteal artery perforators for breasts [80]. Other instances are rarely used flaps or flaps that have been used in the past in reconstructive surgery, each chosen for its unique advantages, though some have been replaced by newer options. For instance, the transverse and vertical rectus abdominis muscle (TRAM and VRAM) flaps were once preferred in breast reconstruction for their substantial tissue volume, but they have largely been replaced by muscle-sparing alternatives, like the DIEP flap, which minimizes donor site morbidity while providing ample soft tissue [81]. Specific flaps, like the medial plantar flap, are frequently reserved for defects requiring glabrous skin, such as hand or heel reconstructions, while toe transfers are commonly employed for finger amputations to restore fine function and appearance [82,83]. Propeller flaps, including the internal mammary artery perforator, lateral intercostal artery perforator, and dorsal intercostal artery perforator flaps, or calf flaps, are utilized for their rotation versatility in trunk and limb reconstructions, though their complexity can limit widespread use [84].

The level of evidence regarding commonly used free flap morbidity is low. RFFF morbidity is predominantly low, with most studies being non-experimental, highlighting a need for more rigorous research [85]. However, there are systematic reviews that emphasize the occurrence of neuropathic pain, which can be a common issue, affecting approximately 23% of patients, while hypoesthesia occurs in about 34% of cases [86]. A systematic review of thigh-based flaps identified paresthesia as the most common donor site complication, with a range of 0–36.4%, followed by wound dehiscence, contour deformity, and pain, indicating that while complications are generally minimal, they are not negligible [87]. For fibula flaps, the perioperative donor site complication rates vary significantly, with reports ranging from 2% to 38% in different studies [88,89]. Long-term complications can involve difficulty walking, pain, and paresthesia in the distribution of the peroneal nerve, as well as joint stiffness and muscle weakness [88]. For latissimus dorsi, a retrospective analysis identified seroma at the donor site as the most common complication, occurring in 26% of cases, with 19% requiring therapeutic intervention [89].

### 3.2. Donor Sites and Their Applications

Upper limb donor sites are primarily characterized by their thin, pliable tissue, making them suitable for intricate reconstructions, particularly in the head, neck, and facial areas. A unique advantage of upper limb donor sites is the consistent vascular anatomy, which offers a dependable blood supply and facilitates a long vascular pedicle, giving surgeons flexibility in positioning. However, flaps from this area are used for small-sized defects, and also, considerations around donor site morbidity are essential; visible arm or forearm scarring and potential sensory deficits can occur [90,91,92].

The trunk and abdomen donor sites, particularly through muscle-sparing and perforator-based flaps, are valuable for reconstructive needs requiring significant tissue volume and robust vascular supply. These areas provide a large quantity of well-vascularized soft tissue and also bone from the scapula or ribs for extensive reconstructions, such as breast and chest wall defects and head and neck reconstruction, where contour and volume are crucial. Donor scars in these areas can often be concealed, which enhances patient satisfaction in terms of aesthetics. However, the technical complexity of perforator dissection requires careful preoperative planning, including imaging to map out vascular patterns to ensure reliable blood flow and minimize complications [93,94,95].

Lower limb donor sites are particularly advantageous when large volumes of durable tissue or bone are required, making them ideal for extremity and mandibular reconstructions. In addition, they allow for a two-team approach for most anatomical areas that need reconstruction. Anatomically, lower limb sites are well supplied by long and reliable pedicles that allow for versatility in positioning. These donor sites, however, vary in thickness, giving surgeons options for thin or bulkier tissues based on reconstructive need. One of the primary advantages of the lower limb is the multitude of flaps that can be harvested containing bone, muscle, and thin or thick skin, making it particularly suited for reconstructions in most areas, but donor site morbidity, such as joint instability or gait changes, can be a concern. Advances in preoperative imaging and meticulous dissection techniques have helped minimize these functional impacts, supporting the use of lower limb donor sites as reliable and versatile sources for complex reconstructions [61,96,97,98].

### 3.3. Donor Site Morbidity: Types and Mechanisms

Donor site morbidity is a significant consideration in reconstructive microsurgery, as the removal of skin, nerves, muscle, or bone from donor sites can result in various degrees of functional, aesthetic, and sensory impacts, as seen in Table 2. Morbidity types can range from acute postoperative complications, such as wound healing delays and infections, to long-term effects, like muscle weakness, reduced range of motion, and chronic pain. The severity and nature of morbidity are closely related to the flap type and the anatomical structures involved; for instance, muscle-sparing techniques tend to reduce functional deficits, whereas flaps harvested from highly visible areas may lead to greater aesthetic concerns. Advances in flap design and surgical techniques have mitigated some of these impacts, yet donor site morbidity remains a central focus, with preoperative planning aimed at optimizing outcomes while minimizing adverse effects [99,100].

Comparing donor sites across functional, aesthetic, sensory, and chronic pain considerations reveals a complex interplay of benefits and drawbacks that guide flap selection in reconstructive surgery. Functionally, lower limb donor sites, like the ALT and fibula flap, often result in some degree of reduced strength or mobility due to the demands on muscle or bone structures. For example, the fibula flap, despite being a popular choice for mandibular reconstruction due to its robust structural qualities, can result in ankle instability and gait disturbance, as well as permanent peroneal nerve palsy, gait disturbances, and great toe contracture, each occurring in 4.9% of cases [60,133,134,135]. Conversely, the RFFF and MSAP flaps, harvested from the upper limb and lower leg, respectively, are more suited for precise soft tissue reconstruction with minimal impact on functional movement. Studies have shown that while these functional limitations are typically reversible and do not affect long-term daily activities, there is a higher incidence of temporary and persistent numbness, as well as cold intolerance, in patients undergoing RFFF compared to those receiving ulnar forearm free flaps [92,106]. While the RFFF impacts hand function due to grip strength reduction, it remains a valuable option for head and neck reconstructions that demand a thin, pliable tissue profile [92].

Aesthetically, upper limb donor sites, such as the RFFF, are often less favorable due to visible scarring, especially in patients concerned with forearm aesthetics. The forearm scar from the RFFF is highly visible and may deter patients who prioritize appearance, as opposed to the more concealed scars of the LD and DIEP flaps, which are usually hidden beneath clothing [67,136]. The DIEP flap, which spares abdominal muscle and limits contour irregularities, is especially advantageous in breast reconstruction for patients who value aesthetic outcomes and minimal scarring [137]. However, the LD flap may lead to shoulder bulk, which can be aesthetically undesirable for some patients. Despite these limitations, the LD flap is still chosen in cases where a large volume of tissue is needed for chest wall reconstructions, balancing functionality and volume against the aesthetic drawbacks [67].

Sensory changes and deficits present a distinct set of considerations, with donor sites like the ALT and RFFF resulting in notable sensory loss at the harvest site. While the ALT flap offers a reliable, large tissue volume for complex reconstructions, patients can experience persistent thigh numbness with sensory changes [138]. The RFFF, due to its forearm location, has a higher incidence of sensory deficits, with patients frequently reporting numbness or altered sensation, which impacts daily activities and patient satisfaction [86]. On the other hand, donor sites, like the SCIP and MSAP flaps, result in minimal sensory impact, largely due to their positioning and the smaller nerve networks in these areas. These sensory advantages make the SCIP and MSAP favorable for smaller, more aesthetically sensitive reconstructions where sensation preservation is a priority [44,73].

Chronic pain is a critical long-term consideration. The LD flap, while effective in providing bulk, is associated with a notable incidence of shoulder pain, which can persist due to its impact on shoulder stability and mobility [131]. Similarly, abdominal donor sites, like the DIEP flap, may lead to chronic pain in some patients, often linked to abdominal nerve sensitivity, although this is still a preferable alternative to the more invasive TRAM flap, which is prone to hernia formation [76,139]. Interestingly, the fibula flap, despite involving bone harvest, shows relatively low levels of chronic pain, which, combined with its durability, makes it a favored choice for mandibular reconstructions where pain management and functional stability are priorities [26].

The choice of donor site is often a balance between the specific reconstructive requirements, patient priorities, and the known comorbidities associated with each flap. Flaps with higher functional or sensory morbidities, like the ALT or RFFF, are often selected for their adaptability and ability to meet complex reconstructive needs, despite some disadvantages. Conversely, donor sites like the SCIP, DIEP, and MSAP are chosen for their minimal sensory and aesthetic impact and are suitable for cases where scarring and sensation are paramount concerns. Ultimately, the strategic selection of a donor site is guided by an understanding of these comparative advantages, allowing surgeons to align flap choice with the unique goals and expectations of each patient [92,94,98].

### 3.4. Impact of Donor Site Morbidity on Quality of Life

QoL assessments in microsurgery are increasingly essential, as they provide a validated means of evaluating the long-term effects of donor site morbidity across physical, psychological, social, and occupational domains. Tools, such as the SF-36 Health Survey and the EQ-5D, are validated in microsurgery for their reliability in capturing patient-reported outcomes related to physical function, pain, and mental well-being [140,141,142].

For fibula flap donor sites, the Star Excursion Balance Test (SEBT) and the Foot and Ankle Disability Index (FADI) are utilized to assess ankle function and overall lower leg functionality, respectively, indicating minimal limitations post-surgery [24]. In the context of radial forearm free flap (RFFF) donor sites, the EuroQol 5D is used to measure QoL, alongside tools like the Doleur Neuropathique 4 and visual analog scale for pain and the Patient-Reported Wrist and Hand Evaluation for hand function, highlighting the significant impact of neuropathic pain on QoL [143]. Morphometric measurements have also been proposed for risk assessment of donor site complications in abdominally based breast reconstructions, offering a personalized approach to enhance postoperative satisfaction and QoL [144]. Additionally, the University of Washington Quality of Life (UW-QOL) questionnaire is applied to compare outcomes between ALTs and RFFs, showing better QoL and fewer morbidities with ALTs [145]. In addition, the Patient and Observer Scar Assessment Scale (POSAS) has been used to subjectively assess donor site scars in both the fibula and anterolateral thigh flap reconstructions, highlighting the importance of aesthetic outcomes [146,147].

Donor site morbidity is a significant factor influencing the QoL outcomes for patients undergoing reconstructive surgery, as it can affect physical, psychological, and social well-being long after the initial surgery, as seen in Table 3. Functional limitations, chronic pain, and sensory deficits from donor sites, like the radial forearm and fibula, can lead to decreased mobility and difficulty with everyday tasks, which impacts a patient’s independence and daily comfort. Additionally, visible scarring, especially on exposed areas, like the forearm, can lead to body image concerns and reduced confidence, which may hinder social interactions and psychological well-being [148,149]. Sensory changes and chronic pain also play a considerable role in QoL, as persistent numbness or discomfort can be distracting or debilitating, affecting sleep, work, and recreational activities. These outcomes emphasize the importance of careful donor site selection and preoperative counseling to ensure patients understand potential morbidities and can make informed choices that align with their priorities and lifestyle [150].

### 3.5. Strategies to Minimize Donor Site Morbidity and Increase QoL

Recent advances in surgical techniques have been instrumental in minimizing donor site morbidity in microsurgery. Innovations in flap harvesting, such as perforator-based techniques and muscle-sparing and nerve-sparing approaches, allow for the transfer of necessary tissue while preserving critical anatomical structures, reducing functional deficits and aesthetic concerns at the donor site. For example, developments in the DIEP flap harvest, which focuses on deep inferior epigastric perforator vessels, avoid abdominal muscle damage, improving outcomes in physical strength and aesthetic appearance postoperatively. In addition, preoperative imaging technologies, including Doppler ultrasound and CT angiography, have refined flap selection and minimized dissection-related complications by helping surgeons precisely map vascular pathways. These advances are crucial for reducing morbidity in complex reconstructions, ensuring that patients can achieve both functional and aesthetic restoration without unnecessary loss of donor site integrity [99,166].

Acute management of donor site complications involves a multi-faceted approach to control pain, promote wound healing, and prevent immediate functional limitations. Postoperative pain management strategies now often include regional anesthesia and local anesthetics, reducing reliance on opioids and enabling earlier mobilization. Effective wound care, such as negative pressure wound therapy, has also been shown to accelerate healing and reduce infection rates at the donor site. In cases where muscle or nerve deficits are anticipated, early physical therapy protocols focusing on range of motion and strength preservation are essential to minimize acute impairments. Additionally, monitoring for early signs of sensory loss or circulatory issues is critical for addressing complications before they progress, ensuring optimal outcomes in the immediate postoperative period [167].

Long-term management of donor site morbidity focuses on addressing chronic pain, sensory deficits, and functional impairments that may arise post-surgery. Chronic pain, often due to nerve involvement or scar tissue, is managed through a combination of physical therapy, nerve modulation techniques, and, in some cases, minimally invasive surgical revisions. Sensory rehabilitation is also key in cases where numbness or altered sensation persists; techniques like sensory re-education and nerve grafting in the case of intraoperative damaged sensory nerves using autologous reconstruction or alloplastic reconstruction are used [168,169,170]. Using these methods has shown promising results in restoring sensation. Functional impairments, such as shoulder weakness from latissimus dorsi harvest or ankle instability following fibula harvest, are managed through targeted strength-building exercises and, in cases where mobility is severely affected, orthotic support. Personalized, ongoing rehabilitation programs can significantly enhance long-term outcomes and quality of life, helping patients regain independence in daily activities [162,167].

#### 3.5.1. Surgical Strategies

The development of alternative flap options and new technologies is significantly broadening reconstructive surgery possibilities, providing less invasive solutions with reduced donor site morbidity. Flaps like the MSAP and SCIP flaps are increasingly favored in smaller reconstructions due to their ability to deliver reliable coverage with minimal functional or sensory deficits. These flaps are particularly valuable in cases where delicate tissue coverage is required but where traditional, bulkier flaps could impose unnecessary limitations on movement or sensation. As these options offer targeted reconstructive capabilities with fewer postoperative issues, they are reshaping the landscape of surgical planning for head, neck, and extremity reconstructions [77,92].

Refining surgical techniques, including nerve-sparing and muscle-sparing approaches, has played a crucial role in reducing morbidity and improving outcomes in reconstructive surgery. These techniques aim to preserve critical anatomical structures at the donor site, thereby minimizing the functional limitations often associated with traditional flap harvests. Nerve-sparing methods, for instance, are increasingly employed to retain sensory and motor function by avoiding damage to major neural pathways during flap dissection. Studies have shown that nerve-sparing approaches can significantly reduce sensory deficits and improve postoperative quality of life [171]. Muscle-sparing techniques focus on transferring only the essential tissue layers while leaving underlying muscle intact. This approach not only reduces the physical impact on donor sites, particularly in areas like the abdomen and thighs, but also preserves strength, enabling patients to return to daily activities more swiftly and with fewer limitations [172,173].

Advanced microsurgical techniques, such as perforator flap harvesting, have further contributed to minimizing morbidity by selectively targeting perforating vessels that supply skin and subcutaneous tissues while preserving larger muscle groups. Perforator-based flaps, including the MSAP and TDAP, offer reliable vascularity with reduced donor site morbidity, as they maintain the integrity of the muscle, thereby reducing postoperative pain and preventing long-term functional deficits [77]. Additionally, meticulous intraoperative planning with preoperative imaging, such as Doppler ultrasound and CT angiography, helps surgeons accurately map vascular structures, making it possible to create smaller, more targeted incisions. This approach not only minimizes dissection-related complications but also enhances the precision of the flap design, which is essential for achieving natural contouring in reconstructions of areas such as the head, neck, and extremities [109].

The usage of local flap techniques is invaluable in reconstruction, such as the head and neck area, offering functional and aesthetic benefits while minimizing donor site morbidity. Techniques like the epiglottis flap, used for pharyngeal reconstruction, are particularly useful when primary closure is unfeasible, leveraging spared structures to cover defects without distant harvesting. Similarly, other local options, such as submental flaps, provide versatile solutions for small to moderate defects, especially in carefully selected cases. This principle can be applied to other areas in which local flaps may spare harvesting flaps from other areas, and it is, consequently, a donor site morbidity [174,175].

#### 3.5.2. Non-Surgical Strategies

Preoperative education and individualized planning have emerged as critical components for enhancing QoL in patients undergoing free flap procedures. Comprehensive education on anticipated challenges, such as sensory changes, mobility limitations, and visible scarring, can significantly improve patient preparedness and acceptance of surgical outcomes. Research shows that patients who undergo preoperative counseling tailored to their specific donor site morbidities report lower anxiety levels and greater satisfaction post-surgery [176,177]. Multidisciplinary preoperative planning, involving surgeons, physical therapists, and psychologists, can further optimize outcomes by aligning patient expectations with realistic postoperative results. Involving occupational therapists in the planning stage allows patients to understand adaptations they might need, particularly in cases where flap donor sites impact daily activities or job performance. Such an approach not only empowers patients with practical knowledge but also helps mitigate psychological impacts, ultimately contributing to a smoother recovery and better long-term QoL.

Scar management at donor sites has become a focal area in reconstructive surgery, with advancements in regenerative medicine, stem cell therapies, and fat grafting showing promise for improved aesthetic and functional outcomes. Stem-cell-based approaches, particularly those using mesenchymal stem cells (MSCs), are gaining traction due to their potential for promoting tissue regeneration and reducing fibrotic scarring [178]. MSCs secrete bioactive factors that modulate inflammation and stimulate collagen remodeling, leading to softer, more flexible scar tissue. These cells can be sourced from adipose tissue, bone marrow, or umbilical cord blood, and their application at donor sites has been shown to accelerate healing, reduce scar thickness, and enhance skin elasticity. Clinical studies indicate that MSCs can inhibit fibroblast overactivity, a key factor in hypertrophic scarring, and promote the regeneration of native skin structures, making them a promising addition to scar management protocols in microsurgery [179,180].

Fat grafting, often combined with stem cell therapy, has also emerged as an effective technique for scar management at donor sites. Adipose-derived stem cells (ASCs), which are found in the stromal vascular fraction of fat tissue, are frequently used in fat grafts due to their regenerative properties [181]. When injected into scarred areas, fat grafting not only improves the appearance of scars by restoring volume but also enhances skin quality by introducing ASCs, which encourage tissue remodeling and reduce fibrosis. Fat grafting has been particularly effective in reducing scar contracture and improving skin elasticity in areas with high aesthetic and functional importance, such as the face and limbs. In addition, the integration of ASCs into scar tissue can enhance local vascularization, which further supports healing and tissue flexibility, making fat grafting a preferred approach in microsurgical settings for donor site management [182,183].

In the management of free flap donor sites, Vacuum-Assisted Closure (VAC) or negative pressure wound therapy (NPWT) has demonstrated substantial benefits in reducing postoperative pain, expediting wound healing, and minimizing scar formation. At free flap donor sites, such as the ALT flaps or the lower leg for fibula flaps, VAC therapy stabilizes the wound bed by reducing interstitial edema and enhancing local perfusion, creating an environment that promotes optimal tissue repair. This effect not only accelerates the healing process but also reduces acute postoperative pain by limiting nerve irritation and decreasing wound tension, leading to improved patient comfort and potentially lowering the need for opioid analgesics. It was shown that VAC therapy may lower pain scores in free flap donor sites, which is especially beneficial in sites that are associated with functional demands or patient activity levels [184,185]. Similar findings were reported in a pediatric cohort, noting that VAC therapy minimized pain and supported rapid healing even in complex lower extremity reconstructions, highlighting its utility across age groups [186].

In addition to pain management, VAC therapy has been associated with improved scar quality at free flap donor sites. The controlled negative pressure modulates fibroblast activity and collagen deposition, thereby reducing the risk of hypertrophic scarring and contributing to the formation of flatter, more pliable scars. This outcome is particularly valuable in visible or high-mobility donor sites, where scar flexibility and aesthetic appearance are important for functional and psychological outcomes. Moreover, VAC therapy has shown efficacy in minimizing wound-related complications, such as dehiscence and infection, by creating a sealed wound environment that maintains consistent moisture levels and protects the wound from external contaminants. Recent innovations, such as the integration of silver nanoparticle dressings with VAC therapy, have further enhanced outcomes by adding antimicrobial properties to the wound bed, reducing infection risks, and supporting a more favorable healing environment. In addition, it was demonstrated that combining VAC with silver-based dressings led to improved healing rates and reduced scarring, underscoring the potential of these combined approaches in scar management at donor sites [187,188].

Rehabilitation protocols are essential in post-surgical care for free flap donor sites, with a primary goal of restoring strength, mobility, and functional independence while addressing any procedure-specific morbidities. Rehabilitation is highly tailored to the flap type and the specific donor site comorbidities, focusing on preventing long-term functional limitations and optimizing quality of life. For example, upper limb donor sites, such as the radial forearm, often involve rigorous hand therapy to restore grip strength, dexterity, and fine motor control, which are crucial for daily functions and occupational needs. Lower limb donor sites, like the fibula, typically require intensive gait training and balance exercises to address potential ankle instability and mobility challenges. Early physical therapy is a cornerstone for these cases, emphasizing range of motion and targeted muscle strengthening to offset functional morbidity and promote independence in activities of daily living [189].

In addition to traditional physical therapy, occupational therapy plays a vital role in rehabilitation, particularly in cases where endurance, fine motor skills, or functional independence are compromised. Occupational therapy assists patients in adapting to new physical limitations, enhancing their ability to resume work, hobbies, and daily routines. This is especially important for individuals whose donor sites impact motor functions or involve highly visible scars, which may affect confidence and social interactions. These interventions directly contribute to improved quality of life outcomes by helping patients regain independence and address any psychosocial impacts of the surgery [190].

#### 3.5.3. Emerging Technologies

Emerging technologies, like three-dimensional (3D) bioprinting and tissue engineering, play a crucial role in addressing the challenges associated with donor sites for free flaps. Three-dimensional bioprinting, which involves the layer-by-layer construction of tissues using bioinks composed of living cells, biomaterials, and growth factors, offers a promising alternative to traditional donor free flaps or transplantation methods. Collectively, these innovations are poised to address limitations inherent to traditional flaps, enabling the achievement of reconstructive goals while preserving or even enhancing quality of life for patients [191,192].

Emerging rehabilitation technologies, including virtual reality (VR) and biofeedback, are increasingly being integrated into rehabilitation programs to enhance patient motivation, objectively track progress, and support long-term functional recovery. Virtual reality environments can simulate real-life tasks and settings, which not only help patients regain confidence in their movements but also allow therapists to monitor performance in various scenarios [193,194]. Biofeedback devices provide patients and clinicians with real-time information about muscle activity, helping to refine movements and address specific functional deficits unique to each flap type and donor site. These advancements in rehabilitation technology, combined with structured, flap-specific protocols, are crucial for reducing long-term morbidity and ensuring that patients achieve the best possible outcomes in mobility, strength, and quality of life, ultimately allowing them to fully reintegrate into their personal and professional lives [195].

Additionally, customized post-discharge support, including telemedicine follow ups, offers substantial benefits in managing long-term QoL outcomes. Telemedicine has proven effective for remote monitoring and adjusting rehabilitation plans, especially for patients who may face difficulties in accessing frequent in-person care due to location or mobility limitations. Studies indicate that patients who receive virtual follow-up support experience improved adherence to therapy and reduced risk of complications [196].

In clinical practice, these insights underscore the need for surgeons to base flap selection on both reconstructive and long-term QoL outcomes. Thorough patient counseling on physical, aesthetic, and psychological impacts related to each flap type enables patients to make well-informed decisions, fostering realistic recovery expectations and greater satisfaction with surgical outcomes. By involving patients in a shared decision-making process, surgeons can better address the psychosocial factors influencing recovery, particularly for patients undergoing reconstructions that may have high visibility or functional consequences [197]. Looking forward, further research is needed to develop standardized QoL assessment tools specifically tailored to microsurgical reconstruction, allowing for a more accurate, comparative understanding of donor site morbidity over time. Additionally, advancements in minimally invasive flap techniques and refined preoperative imaging could further reduce donor site morbidity, supporting outcomes that prioritize both aesthetic and functional aspects. Longitudinal studies will be critical in providing deeper insights into the long-term QoL impacts of different donor sites, ultimately guiding future refinements in microsurgical techniques and enhancing our understanding of comprehensive QoL support in reconstructive surgery.

The limitations of this study stem from the constraints inherent to narrative reviews, such as the lack of systematic data extraction and formal evidence grading. They also result from the broad scope of the chosen subject, which encompasses a wide range of reconstructive methods using free flap transfers. Our analysis primarily focused on the most commonly used flaps and generally reported complications, which does not allow for an exhaustive or standardized perspective of the field. Future, more detailed studies could provide additional insights by specifically identifying the complications associated with each type of flap, considering the particularities based on their indications for use. Such research could contribute to formulating more precise recommendations for improving patient recovery and enhancing quality of life. Enhanced recovery after surgery (ERAS) protocols have been successfully applied across various surgical specialties; however, tailored protocols for reconstructive procedures are not yet established. Recent reports encourage this approach in reconstructive microsurgery, including DIEP flap breast reconstruction and treatment of head and neck cancer [198,199,200,201].

## 4. Conclusions

In conclusion, the findings of this review emphasize the critical influence of donor site morbidity on patients’ quality of life following free flap microsurgery. Specific insights highlight the varying functional, aesthetic, and sensory impacts associated with different donor sites, as well as the potential for chronic pain or psychological distress. While advances in nerve- and muscle-sparing techniques, alongside innovations, such as perforator flaps and regenerative therapies, show promise in reducing these complications, challenges remain in standardizing outcome measures and fully addressing long-term implications. A nuanced, patient-centered approach remains essential, balancing the technical demands of reconstruction with a comprehensive understanding of the individual’s well-being. Future research should aim to refine surgical strategies further and enhance rehabilitation protocols, ensuring holistic recovery and improved satisfaction for patients undergoing these transformative procedures.

## Figures and Tables

**Table 1:** Comparison of common free flap options: vascular anatomy, functional components, uses, and key considerations.

Flap	Vessel	Design	Components	Function	Primary Use	Advantages	Limitations	References
Anterolateral Thigh (ALT)	Descending branch of lateral circumflex femoral artery	Fasciocutaneous or myocutaneous	Skin, fascia, muscle, sensory nerve	Soft tissue coverage (neurotized)	Head, neck, extremities	Large, reliable tissue source, easy to contour	Variation of perforators in vascular anatomy	[13,14,15]
Deep Inferior Epigastric Perforator (DIEP)	Deep inferior epigastric artery	Adipocutaneous	Skin, fat	Breast reconstruction	Primarily breast	Muscle-sparing technique, ample volume, aesthetic result	Technically demanding, possible fat necrosis	[16,17]
Radial Forearm Free Flap (RFFF)	Radial artery	Fasciocutaneous	Skin, fascia	Thin coverage	Oral cavity, pharyngeal defects	Thin, pliable, reliable vascular pedicle	Visible donor site, altered sensation, flexor tendon exposure, diminished grip strength	[18,19,20,21]
Fibula	Peroneal artery	Osteocutaneous or osteomyocutaneous	Bone, skin, muscle	Bone reconstruction	Mandible, maxilla, long bone defects	Bone stock and length, can be segmented	Flexor hallucis longus retraction, knee laxity, sensory deficits	[22,23,24,25,26]
Gracilis	Gracilis branch from the medial femoral circumflex vessels	Myocutaneous	Muscle, skin	Functional muscle transfer	Lower extremity, perineal defects	Small muscle, minimal donor morbidity	Limited size, small flap area	[27,28,29,30]
Superficial Circumflex Iliac Artery Perforator (SCIP)	Superficial circumflex iliac artery	Cutaneous	Skin, fascia	Thin coverage	Head, neck, extremities	Minimal donor site morbidity, thin and flexible	Small and short vessel, challenging pedicle dissection	[31,32,33,34]
Latissimus Dorsi (LD)	Thoracodorsal artery	Myocutaneous	Muscle, skin	Volume and bulk	Breast, chest wall	Ample muscle mass, reliable pedicle	Bulky, more prominent donor site scar	[35,36,37]
Thoracodorsal Artery Perforator (TDAP)	Thoracodorsal artery perforators	Fasciocutaneous	Skin, fascia,	Soft tissue coverage	Breast, chest wall	No muscle sacrifice, reliable perfusion	Anatomical variability	[34,38,39,40]
Profunda Artery Perforator (PAP)	Profunda femoris artery	Fasciocutaneous	Skin, fascia	Soft tissue coverage	Lower extremity, breast	Good for thinner patients, easy dissection	Limited tissue volume	[41,42,43]
Medial Sural Artery Perforator (MSAP)	Medial sural artery	Fasciocutaneous	Skin, fascia	Thin soft tissue	Head, neck, limbs	Thin, pliable tissue, low donor site morbidity	Limited surface area	[44,45,46]

**Table 2:** Morbidity and QoL outcomes by flap type: functional, aesthetic, sensory, and pain considerations.

Flap Type	Functional Morbidity (%)	Aesthetic Outcome	Sensory Deficit	Chronic Pain	References
ALT	Acute: Knee extension deficit, limited thigh mobility Chronic: Minimal, but can cause gait disturbance, diminished knee extension force	Visible thigh scar, often tolerable but visible non-aesthetic appearance if covered with STSG Rare contour irregularities	Acute: Thigh numbness Chronic: Persistent mild numbness due to LFCN sectioning	Rare thigh pain	[49,101,102,103,104,105]
RFFF	Acute: Grip strength reduction, wrist stiffness Chronic: Persistent hand weakness, reduced dexterity	Highly visible forearm scar, often a significant aesthetic issue Donor site pigmentation difference	Acute: Forearm numbness, sensation reduction Chronic: Persistent sensory deficit	Persistent pain Cold intolerance	[20,21,92,106,107,108,109]
Fibula	Acute: Ankle stiffness, minor leg weakness Chronic: Ankle instability, gait disturbance, flexion retraction of flexor hallucis longus	Visible leg scar, typically concealed by clothing Potential contour changes along the fibula harvest site	Acute: Lower leg numbness, sensation impact Chronic: Rare persistent numbness	Rare persistent pain	[109,110,111]
DIEP	Acute: Abdominal wall weakness, difficulty with core function, seroma formation Chronic: Occasional hernia formation	Minimal abdominal scarring, hidden below the clothing line Occasional contour irregularities	Acute: Abdominal numbness Chronic: Mild numbness near the scar	Rare persistent pain	[96,112,113,114]
LD	Acute: Shoulder weakness, limited range of motion Chronic: Persistent strength loss, occasional shoulder atrophy	Back scar, can appear bulky, aesthetic concern Donor site skin pigmentation changes	Acute: Numbness near the harvest site Chronic: Persistent numbness	Shoulder pain	[67,115,116,117,118,119]
SCIP	Acute: Minimal functional impact, stable tissue strength Chronic: Rare long-term functional morbidity Lower limb lymphedema	Minimal groin scar, well hidden Generally good cosmetic results in head/neck reconstructions	Acute: Mild groin numbness Chronic: Minimal sensory loss, rare	Very rare pain	[31,120,121,122]
Gracilis	Acute: Minimal functional impact on the thigh Chronic: No significant long-term deficit, rare muscle atrophy	Inner thigh scar, typically minimal concern Potential for mild contour irregularity	Acute: Thigh numbness Chronic: Rare persistent numbness	Rare persistent thigh pain	[123,124]
PAP	Acute: Thigh adductor weakness Chronic: Minimal long-term functional impact, stable leg strength	Posterior thigh scar, typically well hidden Rare tissue contour changes, minimal aesthetic concern	Acute: Thigh numbness Chronic: Rare residual numbness	Rare chronic thigh pain, pain during sitting	[125,126,127]
MSAP	Acute: Calf weakness, minor gait impact Chronic: Rare calf weakness, no significant functional deficit	Posterior calf scar, usually well concealed with low aesthetic impact, hidden under clothing Non-aesthetic scar may remain left at the donor site, especially if a skin graft had to be used	Acute: Mild calf numbness Chronic: Very rare persistent numbness	Rare persistent pain	[128,129,130]
TDAP	Acute: Shoulder stability impact (minimal) Chronic: Rare persistent weakness or range of motion limitation	Back scar, often concealed but occasionally visible Minimal bulk, low aesthetic concern	Acute: Mild numbness near the harvest site Chronic: Rare residual numbness	Rare residual pain	[131,132]

**Table 3:** Quality of life impacts and mitigation strategies by domain in donor site morbidity.

QoL Domain	Impact Factors	Examples by Donor Site	Mitigation Strategies	References
Physical QoL	Pain, Mobility Limitations	RFFF: Forearm pain and grip strength reduction. Fibula: Ankle stiffness and occasional gait changes	Physical therapy, post-surgical rehabilitation, and targeted pain management to restore grip and mobility	[97,151]
	Sensory Deficits	ALT: Thigh numbness, common after large tissue harvest	Preoperative sensory nerve mapping, nerve-sparing techniques, and counseling on potential sensory impacts	[152,153]
	Muscle Weakness	LD: Shoulder weakness, limits upper body strength	Strength training, physiotherapy, and occupational adaptations for tasks requiring upper body strength	[117,154]
Psychological QoL	Body Image Concerns	RFFF: Highly visible forearm scar leading to body image concerns. ALT: Bulkiness and a large thigh scar may concern some patients	Scar reduction treatments and psychological support	[3,13,155]
	Self-Esteem and Confidence	DIEP: Hidden scar, yet self-esteem is impacted if numbness interferes with comfort	Counseling on aesthetic outcomes, offering concealment options, and psychological counseling	[156,157]
	Chronic Pain	RFFF and ALT sensory nerve pain, involvement	Early pain intervention, nerve monitoring, and long-term follow up to manage chronic pain risks	[158]
Social QoL	Social Confidence	RFFF: Visible forearm scar affects social confidence in some cases	Counseling on visible scarring, reassurance, and follow up for body image concerns	[21]
	Participation in Activities	LD: Shoulder weakness during climbing	Encouragement to resume physical activities as tolerated; physiotherapy to enhance comfort with movement	[159,160]
	Visible Scarring	LD: Back scar is often concealed, but it may be a concern for women’s clothing	Guidance on clothing options, scar management for visible areas, and reassurance	[161]
Occupational QoL	Functional Limitations	ALT: Knee extension deficit. Fibula: May affect drivers due to ankle stiffness	Occupational therapy, ergonomic adjustments, and reassignment to lighter duties as needed	[99,162]
	Sensory Impacts Affecting Dexterity	RFFF: Reduced dexterity impacts jobs needing fine motor skills. Fibula: Ankle instability may impact standing-based jobs	Pre-surgical consultation about dexterity limitations and early hand therapy post-surgery	[20,97,163]
	Pain or Weakness During Physical Tasks	DIEP: Abdominal pain affects physical exertion in jobs with lifting. PAP: Limited mobility restrictions, ideal for non-labor-intensive roles	Job reassignment and restrictions on lifting for the DIEP; flexibility in roles to reduce strain	[164,165]

## Data Availability

Not applicable.
